# Does impaired cardiac function affect peripheral arterial angioplasty outcomes?

**DOI:** 10.1186/1749-8090-10-S1-A114

**Published:** 2015-12-16

**Authors:** Kok H Yap, Yew L Woo, Anna LW Tam, Phong T Lee, Yi C Tham, Nern H Kao

**Affiliations:** 1 Department of General Surgery, Changi General Hospital, Singapore, Singapore, 529889, Singapore; 2 Department of Cardiothoracic Surgery, National Heart Centre Singapore, Singapore, 169609, Singapore

## Background/Introduction

Peripheral vascular disease is commonly associated with concomitant coronary heart disease and congestive heart failure.

## Aims/Objectives

We aim to perform a cohort study to evaluate if revascularization of lower limb is sufficient for wound healing in patients with impaired cardiac function.

## Method

We performed a retrospective study in a tertiary vascular surgical centre on patients with elective lower limb angioplasty for critical limb ischaemia from June 2011 to June 2012. 190 patients were included in this study. Pre-operative demographics, comorbidities, post-operative 30-day mortality, amputation and re-intervention data were collected. The cardiac function was defined as poor (≥40%), moderate (40-54%), and good (≤55%). Univariate and multivariate analyses were done to identify the pre-operative morbidities and whether cardiac function affects the outcomes for these groups of patients. The outcomes measured were re-intervention, limb amputation, wound re-debridement, 30-day post-operative mortality.

## Results

Our patient population consisted of 190 patients with 97 males and 93 females. The most common pre-operative morbidity is diabetes (85%). Overall 30-day mortality rate is 5.2%. Poor cardiac function (p = 0.013) and ischaemic heart disease (0.032) were significantly associated with post-operative mortality in univariate analysis. In multivariate analysis, poor cardiac function showed significant association with post-op mortality (p = 0.045). Below knee angioplasty was significantly associated with the need in amputation in univariate (p = 0.014) and multivariate analyses (0.02).

## Discussion/Conclusion

Poor EF is associated with post-angioplasty 30-day mortality. Below knee angioplasty appears to be associated with higher amputation risk. This is the first evidence in the literature that show the association between impaired heart function and adverse outcome after lower limb angioplasty for critical limb ischemia.

